# The Impact of Human Microbiotas in Hematopoietic Stem Cell and Organ Transplantation

**DOI:** 10.3389/fimmu.2022.932228

**Published:** 2022-07-07

**Authors:** Tirthankar Sen, Rajkumar P. Thummer

**Affiliations:** Laboratory for Stem Cell Engineering and Regenerative Medicine, Department of Biosciences and Bioengineering, Indian Institute of Technology Guwahati, Guwahati, India

**Keywords:** human microbiota, organ transplantation, hematopoietic stem cell transplantation, kidney transplantation, lung transplantation, liver transplantation, heart transplantation, fecal microbial transplantation

## Abstract

The human microbiota heavily influences most vital aspects of human physiology including organ transplantation outcomes and transplant rejection risk. A variety of organ transplantation scenarios such as lung and heart transplantation as well as hematopoietic stem cell transplantation is heavily influenced by the human microbiotas. The human microbiota refers to a rich, diverse, and complex ecosystem of bacteria, fungi, archaea, helminths, protozoans, parasites, and viruses. Research accumulating over the past decade has established the existence of complex cross-species, cross-kingdom interactions between the residents of the various human microbiotas and the human body. Since the gut microbiota is the densest, most popular, and most studied human microbiota, the impact of other human microbiotas such as the oral, lung, urinary, and genital microbiotas is often overshadowed. However, these microbiotas also provide critical and unique insights pertaining to transplantation success, rejection risk, and overall host health, across multiple different transplantation scenarios. Organ transplantation as well as the pre-, peri-, and post-transplant pharmacological regimens patients undergo is known to adversely impact the microbiotas, thereby increasing the risk of adverse patient outcomes. Over the past decade, holistic approaches to post-transplant patient care such as the administration of clinical and dietary interventions aiming at restoring deranged microbiota community structures have been gaining momentum. Examples of these include prebiotic and probiotic administration, fecal microbial transplantation, and bacteriophage-mediated multidrug-resistant bacterial decolonization. This review will discuss these perspectives and explore the role of different human microbiotas in the context of various transplantation scenarios.

## Introduction

The human body is home to a vast and immensely complex ecosystem of bacteria, fungi, archaea, helminths, protozoans, parasites, and viruses, collectively referred to as the human microbiota (1–3). In fact, it is estimated that the ratio of human cells and microbial cells constituting the human body is approximately 1:1 (4). Moreover, the human gut metagenome is approximately 150 times larger and contains two orders of magnitude more unique genes than the human genome (5). Although the human microbiota hosts a diverse variety of microbial life spanning all three domains of life (bacteria, archaea, and eukarya), bacteria outnumber other microorganisms by up to three orders of magnitude (4). It is estimated that between 500 and 1,000 unique species of bacteria inhabit the human body at any given point in time (6).

The gut microbiota is the most diverse, studied, and densest microbiota in the human body. Apart from this, several other locations of the human body such as the oral mucosa, respiratory tract, genitals, and the ocular surface host their own unique microbiotas (2, 4, 7–11). Each of these microbiotas possesses its own intricacies and idiosyncrasies. What is even more interesting is that several unique insights pertaining to health and disease can be uncovered from analyzing each of these microbiotas in isolation and in combination with each other. In fact, studies accumulating for the past several years suggest that the human microbiotas can be decomposed into their distinct constituent subcomponents such as the mycobiome (the fungal and yeast components of the microbiota), virome (the viral component of the microbiota), and even the phageome (the bacteriophage component of the microbiota), each contributing to the overall nature of the community-level interactions the constituent microbiota has with host physiology (12–14).

**Figure 1:**
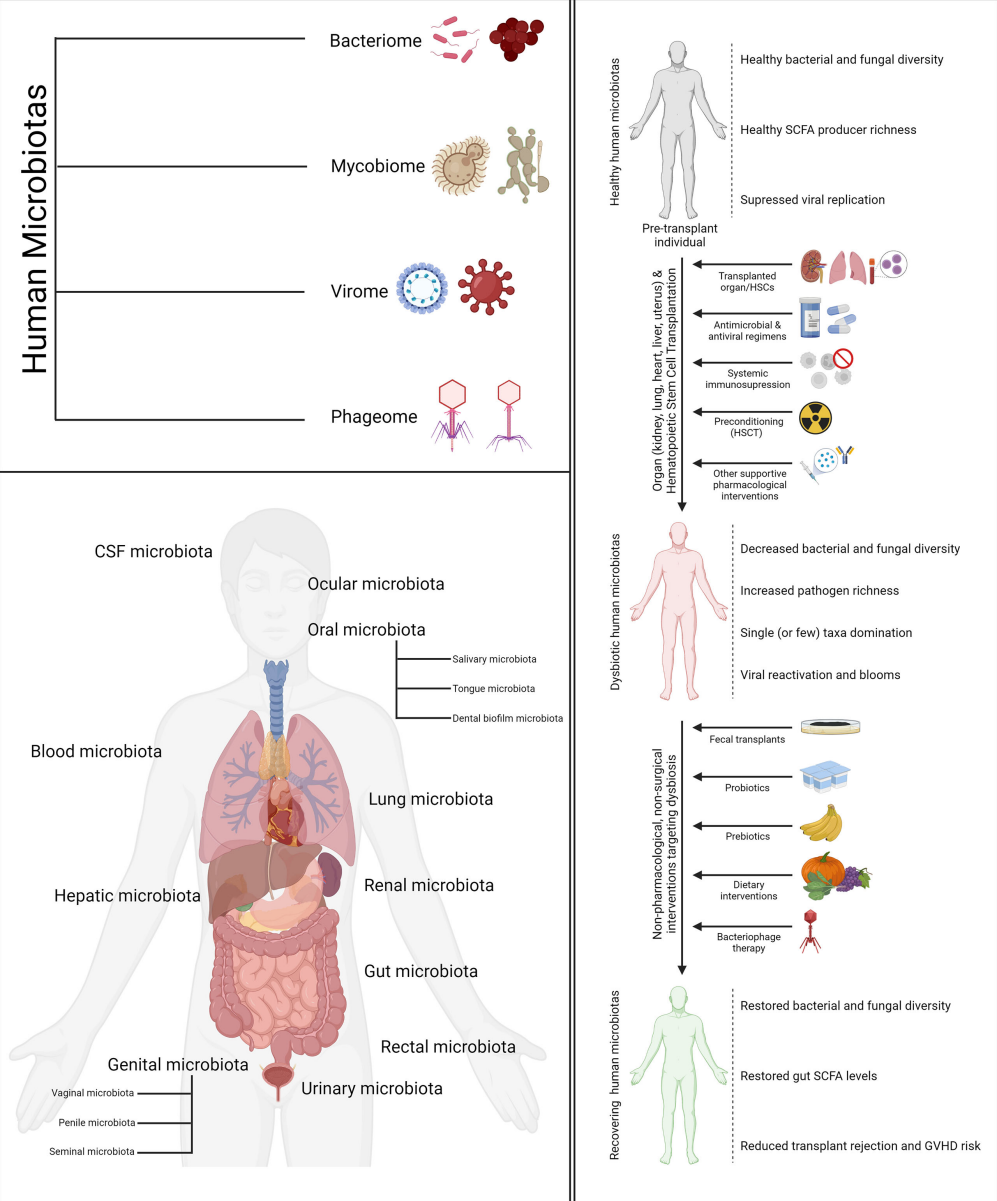
Types, location, and relationship with transplantation and human health. The four components of the human microbiomes discussed in the review (*top left*). The various human microbiotas and their location mentioned in the review (*bottom left*). The impact of cell and organ transplantation on the various human microbiotas and non-pharmacological interventions to ameliorate the same (*right*).

Recent developments in the field of metagenomics and high-throughput sequencing have potentiated a fast-growing body of research that suggests that dysbiosis may play a significant role and act as a biomarker for a host of aberrant pathological conditions. Broad-scale community-level changes in the diversity and composition of a microbiota, which often underlie a diseased or perturbed physiological state, are referred to as dysbiosis. The human immune system is one of the key regulators as well as modulators of the human microbiotas. There is adequate evidence suggesting that a complex choreography between the commensal microbiota and the mammalian immune system plays critical roles in the development of the innate and adaptive immunity as well as the maintenance of host-microbe symbiosis (15). This complex choreography is also one of the key determinants as well as modulators of transplant success and host vs. graft disease in hematopoietic stem cell transplantation (HSCT) and organ transplantation. The following sections will explore the importance of different human microbiotas in the context of stem cell and organ transplantation. A visual summary of the components of most human microbiotas (bacteriome, phageome, virome, and mycobiome), the various human microbiotas discussed in this review, and how transplantation, dysbiosis, and therapeutic interventions targeting the two come together in the big picture is illustrated in **Figure 1**.

## Hematopoietic Stem Cell Transplantation

Hematopoietic stem cell transplantation (HSCT) is a clinical procedure involving the infusion of healthy donor multipotent hematopoietic stem cells (HSCs) to correct a patient’s damaged/defective bone marrow/immune system and consequently reestablish a normal hematopoietic function (16, 17). It is a standard course of treatment for a variety of fatal malignant and non-malignant blood disorders such as leukemias, severe aplastic anemia, multiple myeloma, immune deficiency disorders, and lymphomas (18–24). Broadly speaking, HSCT has three clinical-use cases. Firstly, HSCT enables the replacement of malignant or genetically defective HSCs with healthy fully functional stem cells. Secondly, HSCT resuscitates the depleted HSC population in patients receiving high-dose radiation and/or chemotherapy, thus providing them with a chance of survival and recovery. Finally, in the case of certain cancers (such as leukemia), HSCT provides the patient with a readily available pool of immunocompetent cells capable of eradicating malignant cells. This is referred to as the graft-versus-leukemia effect (25, 26).

According to a retrospective evaluation of worldwide HSCT activity trends conducted by the Worldwide Network for Blood and Marrow Transplantation (WBMT), more than 1,298,897 HSCT procedures were carried out between 1957 and 2016. WBMT is an association of societies involved in cellular therapies (stem cell transplantation, stem cell donation, and cellular therapy) with the mission of promoting excellence in HSCT (27, 28). In 2016 alone, 89,070 HSCT procedures were conducted across 1,662 centers across the globe (29). Although HSCT is the oldest cellular therapy for hematologic malignancies, it remains, to this day, a high-risk procedure. A number of transplantation-related complications such as acute as well as chronic graft-versus-host disease (GVHD), malignancy relapses, conditioning-related toxicity, immunodeficiency, and opportunistic infections are perplexing causes of high morbidity and mortality among allogeneic HSCT recipients (26).

HSCT can be of two types: autologous and allogeneic (25). In autologous transplantation, the stem cells are harvested and transplanted back into the same individual. In allogenic transplantation, the stem cells are harvested from an immunologically compatible healthy donor (typically sourced from the public *via* a national registry) and transplanted into a patient. Since the donor and recipients are one and the same, autologous HSCT does not require HLA-matched donors, poses no risk of GVHD, and does not require immunosuppressive therapy. GVHD is a systemic disorder that occurs when a graft recipient’s body cells are recognized as foreign entities and attacked by the graft’s immune cells (30, 31). It is the most well-recognized complication observed in HSCT recipients (32). Autologous transplantation, therefore, presents a much lower risk of post-treatment life-threatening complications and opportunistic infections. Moreover, for autologous HSCT, treatment-related mortality has been documented to be lower than 5% in most studies (25). Furthermore, elderly and other high-risk patients can tolerate autologous HSCT relatively well (33–36). Autologous HSCT, however, has its drawbacks as well. For example, a transplanted population of autologous HSCs may inadvertently contain clonogenic tumor cell populations, which can contribute to relapse (25). Allogeneic HSCT finds application predominantly in the treatment of leukemias and myelodysplastic syndromes, whereas autologous HSCT is documented more commonly in patients with solid tumors, lymphomas, and myelomas (25).

Dramatic compositional changes in the gut microbiota are observed because of HSCT. Many groups have expressed interest regarding the nature of these changes for their potential applicability as the biomarkers of HSCT outcomes and early indicators of patient treatment response. Several compelling studies have been reported in this direction (37–43). For example, in one very comprehensive study, weekly stool samples from 66 HSCT-recipients were collected and the relative abundance of bacterial taxa was analyzed starting pre-transplant and continuing weekly until 100 days post-transplant (38). The authors observed an association between the GVHD risk and decreased alpha diversity of the stool microbiota (38), a finding in agreement with other similar studies (39, 42, 44). Furthermore, the presence of some species belonging to the *Bacteroides* genus was found to be positively correlated with GVHD (*B. dorei)*, whereas others were negatively correlated (*B. ovatus, B. caccae*, and *B. thetaiotaomicron*), possibly due to varying metabolic, virulent, and inflammation-promoting capabilities. This study was the first to identify several species of *Actinobacteria* and *Firmicutes*, typically found in the oral microbiota, to be positively correlated with subsequent severe GVHD development (38). In the same year, another study profiling the intestinal microbiota of 541 patients found similar associations in certain bacterial groups constituting the intestinal flora (*Eubacterium limosum*, *Anaerofustis stercorihominis*, *Pseudoramibacter alactolyticus*, and *Peptococcus niger*) and disease relapse/progression within 2 years following allogenic HSCT (41). A recent observational study analyzed the microbiota composition of 8,767 fecal samples obtained from 1,362 allogeneic HSCT recipients distributed across four centers. The gut microbiota diversity losses and patterns of single taxa domination (most commonly *Enterococcus* and *Streptococcus*) were observed to be consistent across all four transplantation centers, although a fair degree of heterogeneity was observed among the transplant recipients; not all patients displayed the same patterns. Furthermore, the authors identified geography-invariant associations between decreased gut microbiota diversity and elevated transplantation-related as well as GVHD-related fatal complications (44).

Aside from microbe-based biomarkers, the search for novel biomarkers indicative of HSCT outcomes in the gut microbiota has also yielded interesting biochemical and histological biomarkers such as decreased urinary 3-indoxyl sulfate levels and reduced Paneth cell counts (40, 43). Paneth cells are a type of specialized secretory epithelial cells typically found in the small intestinal crypts of Lieberkühn. They are known to play a role in gut microbiota regulation by secreting antimicrobial peptides such as α-defensins and the antimicrobial lectin RegIIIγ (45). Dietary protein–derived tryptophan is degraded to indole by the intestinal microbiota, which is subsequently oxidized and sulfonated in the liver to form 3-indoxyl sulfate. Microbiota-derived indole and its derivatives are critical for the maintenance of the human gut microflora due to their bacteriostatic, antifungal, epithelial function–regulatory and inflammation-modulating properties (40, 43, 46). In another study, a group analyzed the fecal microbiota metabolites of 44 hematologic cancer patients before undergoing HSCT up to 100 days post-transplant. They reported a correlation between fecal indole and butyrate concentrations with post-transplantation gut microbiota diversity. For example, fecal samples enriched with *Clostridiales* had high butyrate levels whereas *Bacteroidales* enrichment was associated with high indole levels. This study thereby demonstrated the potential applicability of bacterial metabolites as surrogate markers of microbial diversity and the enrichment of specific taxa, which could, in turn, provide vital insights about transplant outcomes (47). In fact, the influence of microbial metabolites such as tryptophan, butyrate, propionate, hexanoate, isobutyrate, riboflavin, 2-propanol, acetaldehyde, dimethyl sulfide, isoprene, and riboflavin, on HSCT has been explored in both human and mouse models by several different groups in considerable detail (48).

As plenty of evidence is accumulated regarding the crucial role the gut microbiota plays in health, disease, immunity, and post-HSCT recovery, rethinking standard clinical practices has become essential. One group compared parenteral nutrition with enteral nutrition in the context of compositional and functional recovery of the gut microbiota in pediatric HSCT patients (49). Enteral nutrition is a modality of clinically assisted nutrition and hydration in which nutrition in the form of a normal oral diet or liquid supplements is delivered directly to the small intestine/stomach. Parenteral nutrition, on the other hand, is another modality of clinically assisted nutrition and hydration in which liquid nutrients (carbohydrates, proteins, fats, vitamins, minerals, and electrolytes) are delivered straight into the circulation by completely bypassing the gastrointestinal (GI) tract, thereby decoupling food ingestion with food–gut microbiota interactions. During the post-HSCT recovery stage, the gut microbiota structure of the enteral nutrition–fed individuals were observed to gradually restore to a pre-HSCT structure. For example, the relative abundance of several bacterial species belonging to the genera known to populate the gut microbiota, such as *Faecalibacterum, Dorea, Blautia, Bacteroides, Parabacteroides*, and *Oscillospira*, were observed to stabilize in enteral nutrition–fed individuals. Similar observations were, however, not made in the gut microbiota of the parenteral nutrition–fed individuals as a comparable recovery endpoint was never achieved. Furthermore, the fecal concentration of small-chain fatty acids such as butyrate was restored to pre-HSCT levels within 4 months of the procedure only in enteral nutrition–fed individuals (49). This observation is of considerable interest as intestinal butyrate levels and, consequently, the presence of butyrate-producing strains in the intestine has been documented to improve intestinal epithelial cell junction integrity, decrease apoptotic activity, and mitigate GVHD (49, 50).

A fast-growing body of research has emerged over the past several years exploring various gut microbiota modulation strategies to improve HSCT outcomes and stabilize HSCT-associated gut dysbiosis. The first clinical trial involving the probiotic supplementation of HSCT patients with *Lactobacillus rhamnosus* GG, one of the most widely used probiotic strains, was reported in 2017. Unfortunately, the trial was prematurely aborted as no appreciable probiotic-induced changes were observed in the gut microbiota or on the GVHD incidence rates of enrolled HSCT recipients (51, 52). Moreover, the safety of probiotic consumption by HSCT recipients is a matter of contention. Since HSCT recipients experience chemotherapy- and radiotherapy-induced damage to the gut epithelia, they are at an elevated risk of developing sepsis and bacteremia (53–57). For example, Koyama and colleagues report the cautionary tale of a 54-year-old male acute promyelocytic leukemia patient who consumed probiotic-enriched yogurt and developed septic shock due to the *L. rhamnosus* GG present in the yogurt (54). Other studies, however, have attested to the safety of probiotic supplementation in HSCT recipients. For example, a study aiming to evaluate the feasibility and safety of a 3-week *L. plantarum*–based probiotic supplementation regimen in children and adolescent allogeneic HSCT recipients reported no unexpected adverse effects such as bacteremia (58). Another study analyzing the blood cultures of 3,796 HSCT recipients for signs of bloodstream infections occurring within 1 year of the procedure caused by common probiotic organisms such as *Lactobacillus* species, *Bifidobacterium* species, *Streptococcus* species, and *Saccharomyces* species, found only 0.5% of the cohort to be positive for the same. *Lactobacillus* was the most identified species in the positive blood cultures. On the other hand, no occurrences of *Bifidobacterium* species or *S. thermophilus* were identified (59). Another study probing the safety of *L. rhamnosus* GG–based probiotic administration in a high-risk pediatric HSCT patient cohort also reported no occurrences of bacteremia involving *Lactobacillus* and therefore endorsed the safe use of probiotics even in high-risk HSCT patients (57).

In comparison to probiotics, prebiotics are viewed as a safer dietary intervention since they involve the consumption of gut bacteria–fermentable dietary fibers such as starches, fructooligosaccharides, and galactooligosaccharides, which alters the community structure of microbiota without elevating the risk of bloodstream infections (53, 60, 61). A combination of glutamine, fiber, and oligosaccharides (GFO) constitutes a commercial Japanese enteral supplementation product considered to be beneficial for restoring chemotherapy-induced GI mucosa damage (62, 63). A group performed a retrospective study to verify the validity of this claim. The administration of the prebiotic combination did, in fact, demonstrate mucosa-protective properties and increase short-term survival rates following HSCT. The severity of post-chemotherapy diarrhea was also notably reduced in the prebiotic-supplemented group. No positive impact on the GVHD relapse rate was, however, observed. It is also interesting to note that a *Lactobacillus*-based probiotic supplement was also provided to all the prebiotic-supplemented patients, suggesting the potential existence of a prebiotic–probiotic synergy (63). More studies are, however, necessary to understand how to optimally introduce prebiotic- and probiotic-based supplementation protocols in a standard pre/post-HSCT care regimen. In another study, a resistant starch-and-GFO mix was administered to allogenic HSCT recipients starting from pre-transplantation conditioning to 28 days post-transplant. Resistant starch refers to a broad category of several structurally different starches, all of which resist digestion by human enzymes, thereby surviving transit to the colon where they are fermented by the resident microbes. It is considered to be beneficial for gut health as it promotes the production of butyrate in the gut (64, 65). Prebiotic intake was marked by a decrease in the incidence of acute GVHD, the maintenance of microbial diversity in the gut within brackets and a decreased duration of oral mucositis and diarrhea (66). Oral mucositis, characterized by tissue swelling, is a common complication of anticancer therapy that can impact up to 90% of cancer patient populations (67). The role of nutritional interventions such as optimized energy and protein intake in modifying bacterial diversity, improving allogenic HSCT-recipient survival, and reducing acute GVHD risk has also been explored. A recently published secondary analysis of a randomized, controlled, nutritional intervention trial failed to find any significant impact of nutritional interventions on the gut microbiota, acute GVHD risk, markers of gut barrier functions, or fecal short-chain fatty acid levels of allogenic HSCT recipients (68).

Aside from dietary interventions, fecal microbiota transplantation (FMT) is another gut microbiota modulatory intervention that has been explored in the context of a variety of disease processes and therapeutic interventions. FMT is the administration of a fecal matter solution into the intestinal tract of a recipient to promote microbial community–level changes in the recipient’s gut microbiota. Over the past seven years, multiple retrospective and prospective, and one randomized controlled trial reporting fecal transplantation in the context of HSCT with varying levels of success have been demonstrated (56). The first successful FMT procedure was conducted in 2012 in an immunocompromised HSCT recipient suffering from a severe *C. difficile* infection. Interestingly, standard pharmacologic regimens had failed to provide any symptomatic relief to the patient, but within 2 days of undergoing the FMT procedure, the patient’s symptoms were abated (69). Soon thereafter, another successful case of FMT was reported by another group (70). HSCT recipients suffering from *C. difficile* infections have, in general, responded well to fecal transplantation, although not all patients receiving the treatment have experienced comparable benefits. For example, in one such study, seven HSCT recipients were administered FMT, out of which five were still undergoing immunosuppressive therapy. After the transplant was administered, none of the patients demonstrated any adverse reactions and only one patient experienced an infection relapse, which was remediated by another round of FMT (71). In another report, however, only one of three patients undergoing FMT experienced the successful clearance of a *C. difficile* infection whereas the other two presented with symptomatic recurrence (72). FMT has also been reported to be effective against steroid-resistant acute GVHD in the gut. For example, in one study, three patients experiencing steroid-resistant grade IV gut GVHD received fecal transplants from both related and unrelated donors (73). Although the repeated cycles of FMT were required to bring about sustained symptomatic improvement, two patients experienced a complete resolution of GI GVHD whereas the remaining patient experienced partial resolution (grade I GVHD). Furthermore, as observed in many other studies, the restoration of the depleted gut bacterial richness was associated with clinical improvement in the patients (73). Several studies have reported attempts to induce antibiotic-resistant bacteria decolonization in the gut of HSCT recipients (74–78). In one such study, five pediatric patients were subjected to one course of FMT (sourced from the same donor) prior to allogenic HSCT (78). Although four of five patients tested negative for multidrug-resistant bacterial strains a week after the fecal transplant, only one patient tested negative for the same after a month. Therefore, the study demonstrated that although FMT is a safe and effective approach for short-term multidrug-resistant bacterial decolonization, it is subject to temporal decay (78). The safety of FMT has also been explored in the literature (56). Although most studies have attested to the safety of FMT in HSCT patients, some have reported adverse effects. In one study, a patient who was treated with oral FMT before undergoing allogenic HSCT developed febrile neutropenia and succumbed soon thereafter to extended-spectrum beta-lactamase-producing *Escherichia coli* bacteremia (75). Postmortem blood cultures revealed that the extended-spectrum beta-lactamase-producing *E. coli* strain was introduced into his body *via* the oral FMT capsule. Interestingly, a liver cirrhosis patient enrolled in a different clinical trial (involving the use of FMT for refractory hepatic encephalopathy treatment) also presented with extended-spectrum beta-lactamase-producing *E. coli* bacteremia since both the trials involved the same fecal matter donor (75). Inadvertent FMT-induced viral infections are also a matter of concern. The presence of Norovirus has been reported in stool samples used for FMT that subsequently triggered acute GVHD in an allogenic HSCT recipient. Fortunately, a subsequent virus-free FMT combined with a course of steroids resolved the complications (79). More stringent screening protocols, larger and more geographically diverse clinical trials, and a deeper understanding of the mechanistic underpinnings underlying the clinical findings of FMT trials are required to promote FMT from an experimental line of treatment to mainstream clinical practice.

Since the gut microbiota is the densest and most well-studied human microbiota, it is unsurprising that a significant body of research has found notable associations between changes in the gut microflora and HSCT. However, several interesting connections between HSCT and the human microbiotas aside from the gut have also been reported. There is evidence that the oral microbiota is a contributor as well as severity modulator of oral mucositis in HSCT patients (80–83). Ulceration is a stage of oral mucositis during which there is a high risk of oral microorganisms infiltrating submucosal vessels, leading to bloodstream infections (67, 84). Bloodstream infection is a common albeit serious HSCT complication associated with increased patient mortality (85). Due to the concerning lack of established bloodstream infection–reducing strategies from oral microorganisms, a group conducted a single-center, randomized controlled trial in pediatric HSCT recipients to evaluate whether a xylitol wipe–based intervention could modulate the oral microbiota and decrease the occurrence of infections. The authors reported that the addition of xylitol to a standard oral care regime substantially improved oral health by decreasing the oral pathogen abundance and, consequently, the risk of bloodstream infections from oral microorganisms (7). In addition to the effectiveness of xylitol in reducing bloodstream infections, it has also been reported to reduce dental plaque, gingivitis, and oral ulcerations in patients undergoing HSCT (86). The dental biofilm microbiota is a rich and diverse component of the oral microbiota. It is known to regulate innate oral defenses, communicate with host cells, and modulate immune homeostasis. However, very little is known about dental biofilm dysbiosis in diseased and immunocompromised states (87, 88). Heidrich and colleagues were the first to characterize changes in the dental biofilm microbiota of 30 allogenic HSCT-recipients using high-throughput 16S rRNA sequencing. For this study, supragingival biofilm samples were collected from patients at three distinct phases of the HSCT procedure: before preconditioning, during aplasia, and during engraftment. The patients were subject to standard antibacterial (oral levofloxacin), antiviral (acyclovir or valacyclovir), and antifungal (echinocandins or azoles) prophylaxis as well as pre-HSCT conditioning regimens. Additionally, cephalosporin and antibiotics for anaerobic bacteria (metronidazole, meropenem, or piperacillin/tazobactam) were administered to a subset of patients. The commensal dental biofilm bacteria belonging to the *Streptococcus* and *Actinomyces* genera were observed to decrease alongside increases in potentially pathogenic bacterial genera, such as *Enterococcus*, *Lactobacillus*, and *Mycoplasma*. The high relative abundance of some bacterial species was associated with a decrease (*Veillonella*) in GVHD risk, whereas others (*Streptococcus*, *Corynebacterium*) were associated with an increase. The occurrence of *Enterococcus faecalis* blooms was also strongly associated with an elevated risk of GVHD. A bloom is the sudden expansion of a particular genus from a relative abundance of lesser than 1% during the preconditioning stage to a staggering relative abundance of over 30% at the aplasia or engraftment phase of HSCT (87). This study, therefore, presented evidence suggesting that the dysbiosis of the dental biofilm microbiota, a component of the oral microbiota, may be indicative of a post-HSCT GVHD risk. The dysbiosis of the tongue microbiota, another component of the oral microbiota, of HSCT recipients has also been reported. A group reported an analysis of the tongue microbiota composition of 45 patients suffering from hematological disorders on the day of transplantation. They identified 34 uncommon taxa in the oral cavity, among which the presence of *Staphylococcus haemolyticus* and *Ralstonia pickettii* was significantly associated with an elevated post-transplantation period mortality risk (89). A recent study exploring the correlation between HSCT-recipient oral microbiota changes and treatment outcomes reported comparable findings; a decline in the species diversity of the oral microbiota was associated with elevated relapse risk and increased mortality. Interestingly, in the same study, the diversity of the salivary microbiota, yet another component of the oral microbiota, was found to be uncorrelated with allogenic HSCT outcomes (90). A much older study conducted by a different group reported that the oral microbiota of not all but only the HSCT recipients developing post-transplantation respiratory complications demonstrated notable changes (91). The impact of HSCT on the lung microbiota and its potential implications on therapeutic outcomes is, in fact, an area of growing interest. A group explored the role of the lung microbiota in post-HSCT pulmonary complications using human HSCT recipient-sourced bronchoalveolar lavage samples (92). The lung microbiota was found to correlate with several features of post-HSCT pulmonary complications and was also found to be significantly associated with alveolar inflammatory cytokine concentrations (92). Bronchoalveolar lavage samples from HSCT recipients also demonstrated a significantly elevated relative abundance of *Proteobacteria*. Interestingly, an enrichment of *Proteobacteria* in the gut microbiota has been previously documented to be a major predictor of the development of pulmonary complications in HSCT recipients (92, 93). There is, in fact, evidence that the gut microbiota plays a pivotal role in moderating pulmonary immunity as well as conferring protection against viral and bacterial pathogens (94–96). For example, an enrichment of butyrate-producing gut bacteria has been associated with enhanced resistance to lower respiratory tract viral infections in allogenic HSCT patients (95).

Research exploring the link between the human microbiotas and HSCT has been disproportionately focused on the role of the bacterial microbiota (bacteriome). The role of the virome is understudied and therefore presents a promising area for deeper investigation. A group characterizing the gut virome of 44 HSCT recipients observed the most frequently observed viral families to be Anelloviridae, Polyomaviridae, Picobirnaviridae, and Herpesviridae, among which picobirnaviruses were predictive of GVHD development (12). Moreover, the higher fecal levels of two biomarkers (calprotectin and α1-antitrypsin) associated with corticosteroid response and severity in GVHD were observed in stool samples positive for picobirnaviruses. This is suggestive of a potential connection between picobirnaviruses and gut inflammation. The authors also observed a notable increase of persistent DNA viruses (polyomaviruses, anelloviruses, papillomaviruses, and herpesviruses) alongside a decline of phage richness in the fecal samples of HSCT recipients suffering from enteric GVHD. Interestingly, persistent DNA virus reactivation in the gut was observed in the first 3 weeks after transplantation in individuals who were not experiencing any symptoms of enteric GVHD. In fact, the individuals developing enteric GVHD experienced viral reactivation 3–5 weeks after HSCT, suggesting that GVHD-associated inflammation and/or corticosteroid therapy–mediated immunosuppression may have functioned as a DNA virus reactivation trigger (12, 97). This finding also suggests that persistent DNA virus replication may confer a protective role against gut inflammation and the development of enteric GVHD, as already suggested in inflammatory bowel disease mouse models (12, 97, 98).

The blood and CSF virome have also been explored in the context of HSCT. A group obtained plasma samples from 40 1-month post-transplant allogenic HSCT patients to study the plasma virome. The most frequently detected DNA viruses were polyomaviruses, anelloviruses, herpesviruses, human papillomaviruses, and adenoviruses. The most frequently detected RNA virus family was pegivirus. Interestingly, the human pegivirus was found to be persistent in the allogenic-HSCT blood virome up to a year after transplantation although no associations between human pegivirus infection and HSCT outcomes were uncovered (97, 99). Another group comparing the cerebrospinal fluid virome of patients demonstrating post-HSCT neurological complications with healthy (non-transplantation) controls found elevated levels as well a higher genetic diversity of Torque teno virus (TTV) and related anelloviruses (97, 100). The finding of this study is in agreement with several other more recent studies exploring the suitability of utilizing TTV viral titers as a biomarker for post-HSCT immune function and immunological monitoring (101–105).

The mycobiome has also been investigated in the context of HSCT. In a first-of-its-kind proof-of-concept study, a metagenomic analysis of the gut mycobiota composition of ten children with thalassemia undergoing allogeneic HSCT was done at four different time points (transplant day, 15 days post-transplant, 30 days post-transplant, and 90 days post-transplant) to evaluate how HSCT impacts the diversity of the human gut mycobiota (106). No notable changes in the gut mycobiome were observed up to a month following transplantation, and the dominating phylum was *Ascomycota*. Three months post-transplant, alongside the members of phylum *Ascomycota*, the members of phylum *Basidiomycota* were also observed, although *Ascomycota* was still dominant. Out of the ten children enrolled in the study, three presented with GVHD. The analysis of the gut mycobiota on day 90 revealed that only one child (presenting with acute skin and gut GVHD) had a significantly increased abundance (84%) of *Malassezia restricta* and *M. globosa* while the other two demonstrated no mycobiome alterations. This is an interesting finding as *Malassezia* is among the most abundant members of the skin mycobiota and has been documented to play important roles in the development of skin diseases such as atopic dermatitis, pityriasis versicolor, and psoriasis. Furthermore, the *Malassezia* genus is documented to be among the most abundant genus in human stool samples with *M. restricta* and *M. globose* being the most abundant species within the genus. The mechanistic underpinnings underlying mycobiome dysbiosis or its pathological implications, as observed in the study, are unclear and require further investigation. However, more focused future investigations into the relationship of *Malassezia* spp. and GVHD in HSCT patients may help in the development of novel microbial biomarkers as well as targeted therapeutic interventions involving this genus (106). Although the bacteriome, virome, and mycobiome individually provide interesting insights with potential clinical relevance, a siloed understanding of these individual biomes may not be representative of the community dynamics at play. This is attributed to the fact that the various kingdoms of microorganisms inhabiting the microbiota are engaged in constant crosstalk with each other. Although expansive cross-kingdom microbiota analyses are currently very limited, one such study has been reported by Robinson and colleagues wherein the authors characterized the oral mycobiome as well as oral mycobiome–bacteriome interactions in acute myeloid leukemia patients undergoing remission induction chemotherapy (13). Remission induction chemotherapy aims to reduce the leukemic burden of an individual by means of intensive cytotoxic chemotherapy to ideally achieve a state of complete remission. Following this, further administration of cytotoxic chemotherapy or HSCT is considered to potentially achieve long-term cancer remission (107). In the first longitudinal mycobiome study of its kind, the authors reported the existence of highly dynamic mycobiome–bacteriome interactions and highlighted the need for expansive, holistic studies analyzing interkingdom functional interactions and dynamic changes in the microbial community structure in response to chemotherapy, antibiotic treatment, and their clinical implications (13).

**Figure 2:**
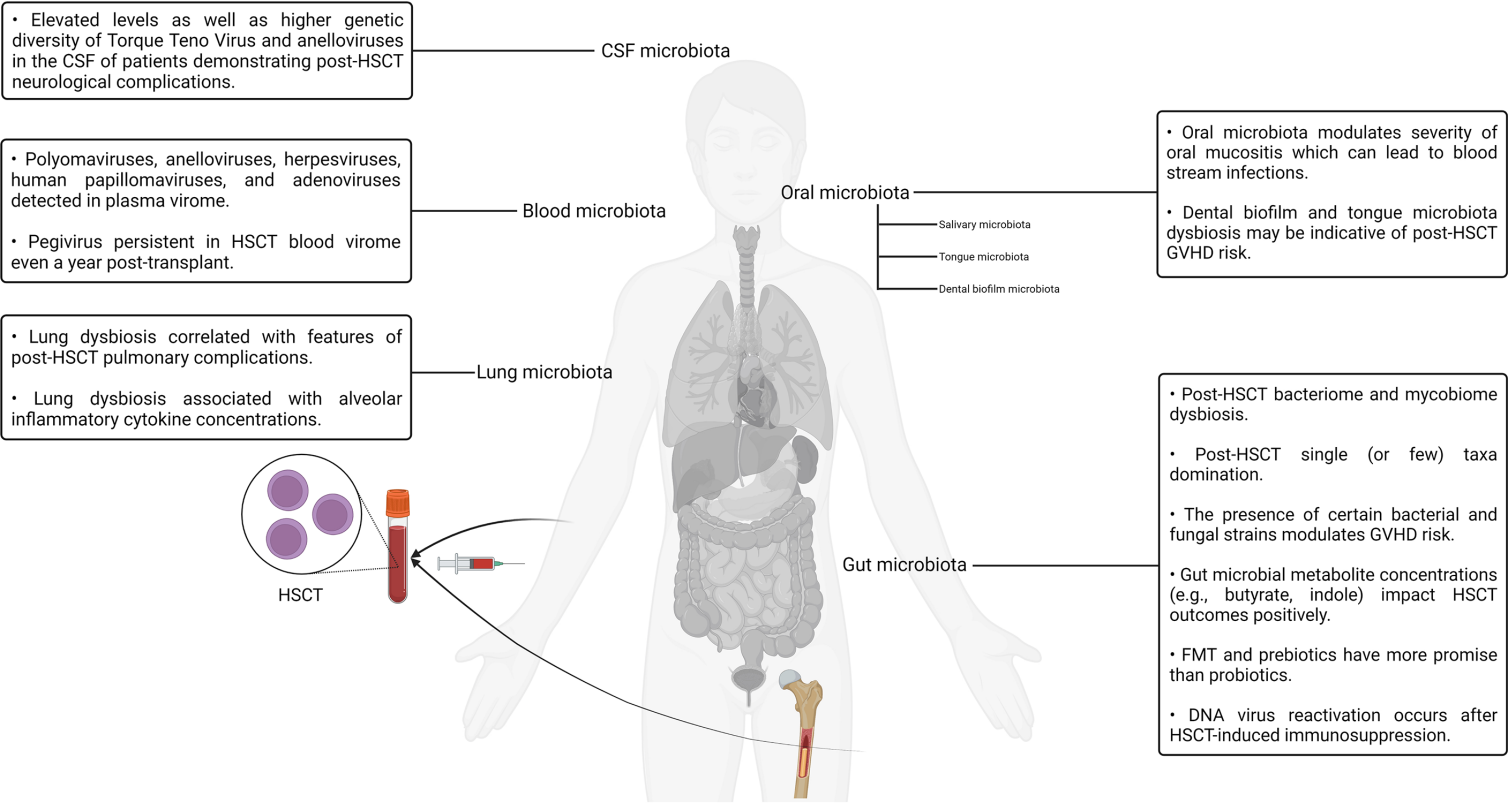
A visual summary of some of the key highlights discussed in the context of hematopoietic stem cell transplantation and the human microbiotas.

Although the interrelationship between stem cells and the human microbiotas is well established in the context of HSCT, other interesting connections have also been reported. It is a well-known fact that the stem cell factor plays a pivotal role in the activation, expansion, differentiation, and survival of mast cells. Furthermore, mast cell progenitors are known to settle in the skin as well as other tissues once they enter the circulation after leaving the bone marrow (108–110). In one study, a predominant component of the bacterial cell wall named lipoteichoic acid was observed to induce the expression of the stem cell factor in keratinocytes. The lipoteichoic acid–stimulated keratinocytes, in turn, effected the recruitment and maturation of mast cells residing within the dermis, thereby establishing a link between the commensal bacterial population of the skin microbiota and mast cell immunobiology (108). The roles played by adipose tissue in modulating the immune system have been explored in the literature for nearly a decade (111, 112). Studies exploring the immunological role of adipose tissue and its interrelationship with the gut microbiota have also surfaced in recent years (111, 112). For example, obesity has been documented to be associated with altered gut microbiota composition as well as aberrant gut barrier function, which, in turn, may potentiate the development of insulin resistance and type 2 diabetes (113). Like the observations reported in the context of the stem cell factor, the stem cell growth factor-beta has been reported to have notable effects on granulocyte/macrophage progenitor cells alongside other cytokines such as the macrophage colony–stimulating factor and granulocyte macrophage colony–stimulating factor (114). A recent study aimed to investigate the presence of any notable associations between the stem cell growth factor-beta, inflammation markers, and insulin resistance in obese male non-alcoholic fatty liver disease or hepatic steatosis patients (114). It was found that C-reactive protein and interleukin-6 levels were predictive of stem cell growth factor-beta concentrations in male patients (114). The role of the gut microbiota has been studied in the context of adipocytes and metabolism regulation. The role of gut microbiota–derived metabolites in regulating host metabolic homeostasis and its contribution to various disease processes have also been investigated (115). For example. one group reported the role of tryptophan-derived gut microbiota metabolites in the regulation of energy expenditure and insulin sensitivity by controlling the expression of a highly conserved miRNA family (*miR-181* family) in the white adipocytes of mice (116). Deeper studies exploring clinically translatable relationships between the host metabolism, immunity, and the resident microflora of the microbiotas are expected to surface in the upcoming years. Our current understanding of the complex interaction patterns between the various human microbiotas and the human body as well as its potential implications for HSCT is still in its infancy. However, as more studies accumulate, significant developments in this domain can be expected in the upcoming years. A visual summary of some of the key highlights discussed in this section is illustrated in **Figure 2**.

## Renal Transplantation

Chronic kidney disease (CKD) is one of the prominent causes of suffering and death around the globe. In the year 2016, CKD was responsible for approximately 1.18 million deaths worldwide, a 200% increase over a span of less than three decades (117). In 2017, over 697 million cases of CKD were reported, out of which approximately 1.2 million patients lost their lives to the disease (118). CKD is classified into five stages based on the severity of the dysfunction and, consequently, the magnitude of clinical interventions required to stabilize the patient. End-stage renal disease (ESRD) is the terminal stage of CKD that is characterized by the cessation of normal kidney function, resulting in the need for renal replacement therapy (RRT) (119, 120). RRT refers to the entire repertoire of clinical interventions aimed at artificially compensating for compromised kidney function in renal failure patients (121). RRT modalities include conservative approaches not involving dialysis, extracorporeal (hemodialysis) and paracorporeal (peritoneal) dialysis, and kidney transplantation (121, 122). Although there is no unified consensus with regard to the number of individuals receiving RRT, with estimates ranging between 1.9 million and 2.6 million for the year 2010, the numbers are expected to rise. In fact, it is estimated that the number of individuals receiving RRT will exceed 5 million by the year 2030 (123–125).

Despite having a higher initial risk of death, kidney transplantation has several advantages over dialysis such as a significantly improved quality of life, a lower risk of long-term mortality, and a decreased risk of cardiovascular events (126, 127). It is, therefore, the best clinical intervention for end-stage kidney failure. Renal allografts are, however, reliant on the lifelong administration of immunosuppressants to suppress T-cell-mediated responses, thereby preventing transplant rejection (128–131). Immunosuppressants like cyclosporine, tacrolimus, and mycophenolic acid revolutionized organ transplantation in the 1980s and 1990s by lowering acute rejection rates and consequently improving short-term graft survival (132, 133). Other drugs such as leflunomide, mycophenolate mofetil, brequinar sodium, and deoxyspergualin also demonstrate a diverse range of inhibitory effects on the immune system and are therefore used as adjunctive therapeutic immunosuppressants. Aside from drugs, monoclonal antibody–based immunosuppressive treatments targeting cytokines, costimulatory signals, cell-surface receptors, and various B-cell epitopes involved in allograft rejection are also used as an adjunct to the maintenance immunosuppression in adult kidney transplant recipients and are commonly referred to as antibody induction therapy (134, 135). For example, alemtuzumab, a humanized CD52-specific antibody, has been shown to achieve a rapid depletion of peripheral and secondary lymphoid T cells as well as B cells, NK cells, monocytes, and dendritic cells in kidney transplant recipients (136, 137). Despite the administration of immunosuppressive treatments, long-term survival rates are unsatisfactory. Approximately 40% of kidney transplants are documented to fail within a decade of transplantation (138, 139). In fact, a study comparing long-term kidney graft survival outcomes in Europe and the United States during the period from 2005 to 2008 found that the 5-year and 10-year graft survival rates among European recipients were 77% and 56%, respectively (140). This is perhaps unsurprising as a constellation of demographic, physiological, and immunological factors alongside clinical interventions such as immunosuppression and prophylactic antimicrobial agents are responsible for determining the outcome of kidney transplantation. Although the clinical repertoire of immunosuppressive treatments has expanded significantly since its inception, the underlying immunomodulatory strategies have mostly remained the same at a mechanistic level.

Given the complex crosstalk that occurs between the immune system and the various microbiotas of the human body, more holistic approaches to immunosuppression and graft rejection prevention are warranted. Over the past several years, there has been a prominent increase in interest surrounding the various human microbiotas and their respective roles in kidney allograft maintenance as well as rejection (141). Furthermore, in addition to the five types of non-invasive chronic kidney transplant rejection biomarker technologies (transcriptomic, cellular, epigenetic, proteomic, and metabolomic) currently utilized, the human microbiota may also become an insightful biomarker to predict allograft outcomes, therapy responsiveness, and patient-specific susceptibilities/sensitivities to specific drugs such as antimicrobials and immunosuppressants (130, 139, 142, 143).

One of the early attempts to evaluate the effect of long-term allograft tolerance–promoting immunosuppression using a high-throughput approach was carried out on the oral bacterial microbiota by Diaz and colleagues (144). The authors noted that although the most commonly and abundantly occurring bacterial species were unimpacted due to immunosuppression, the detection frequency and relative abundance of several bacterial taxa known to behave as opportunistic pathogens in immunocompromised individuals had increased considerably. This finding is in agreement with older reports wherein bacterial species such as *Acinetobacter baumannii, Klebsiella pneumoniae, P. fluorescens, P. aeruginosa, S. aureus*, and *E. faecalis* were observed in the oral microbiota of individuals with weakened immune systems such as hospitalized patients and the bedridden elderly (145). The authors also discussed how differences in administered drugs, drug combinations, and drug dosages between different immunosuppression regimens can impact the resulting oral microbiota changes (144). The impact of immunosuppression on the oral microbiota is, in fact, well documented. A study published as early as 1983 discussed an association between gingival hyperplasia (an oral condition characterized by gum tissue overgrowth) and the immunosuppressant cyclosporin; transplant-driven microbiota changes in immunosuppressed renal transplant recipients can potentiate bacteria-induced inflammation (132, 146). Other studies studying the oral, dental, and periodontal implications of immunosuppression have reported comparable supportive findings. For example, a large study reported that 2 out of every 3 participating kidney transplant recipients had at least one type of oral mucosal ulcer whereas other reports have highlighted the noticeably higher prevalence of oral candidiasis in immunosuppressed patients (132, 147–149). A 31% higher incidence of developing oral lesions was reported in immunosuppressed renal transplant recipients as compared to control subjects in a 2-year long cohort study with a sample size of 100 (150).

Tacrolimus is an immunosuppressant responsible for inhibiting T-lymphocyte signal transduction as well as interleukin-2 (IL-2) transcription and is one of the most common immunosuppressive drugs administered to kidney transplant recipients (141, 151–154). It belongs to a class of immunosuppressive agents referred to as calcineurin inhibitors. Calcineurin is a calcium- and calmodulin-dependent serine/threonine protein phosphatase that dephosphorylates and consequently activates the NFAT transcription factor (155). Tacrolimus forms a complex with the members of a class of proteins referred to as FK506-binding proteins in the cytoplasm, and its immunosuppressive activity is mediated by complexes formed with the FKBP12 isoform, a 12-kDa FK506-binding protein (156, 157). The formation of the tacrolimus-FKBP12 complex results in the inhibition of calcineurin, thereby effecting the downstream deactivation of T-lymphocyte signal transduction as well as IL-2 transcription (157–159). Tacrolimus has a narrow therapeutic range as subtherapeutic levels can lead to immune rejection whereas supratherapeutic doses are nephrotoxic and neurotoxic (160). It is therefore essential to optimize the tacrolimus dosage to minimize the risk of suboptimal transplantation outcomes in kidney allograft recipients. This is, however, a complicated undertaking. Apart from a range of physiological and genetic factors that influence tacrolimus blood concentration in patients, multiple commensal gut bacteria are known to metabolize tacrolimus (161–163). Therefore, intraindividual gut microbiota differences in kidney transplant recipients can potentially result in differential tacrolimus exposure and, consequently, therapeutic efficacy. One insightful murine model–based study has revealed that high-dose tacrolimus treatment results in notable changes in the composition of the gut microbiota denoted by an increase in the beta diversity, a numerical representation of the variation in community composition (141, 154). For instance, compared to the control mice, the tacrolimus-treated mice demonstrated an increase in the abundance of *Bacteroides*, *Allobaculum*, and *Lactobacillus* alongside a concomitant decrease in the abundance of *Oscillospira, Ruminococcus, Rikenella*, and *Ruminococcaceae* (154). It is interesting to note that these genera alongside many others have been reported in diverse yet clinically relevant contexts such as constipation, autism spectrum disorder, and obesity in both murine-based and human studies (164–168). A reduction in numerous microbiota-associated metabolic functions (protein degradation, bioenergetics, xenobiotic breakdown, carbohydrate, and lipid metabolism), some of which are documented to impact immune function (such as glyoxylate metabolism), were also observed in the same study, thereby demonstrating evidence of a complex relationship between immunosuppressant drugs, the gut microbiota, and the immune system (154). Furthermore, mice treated with a combination therapy of low-dose tacrolimus and fecal microbiota transplantation (using fecal matter obtained from a high-dose tacrolimus-treated mouse donor) had a significantly improved allograft survival rate in comparison to mice receiving any one of the interventions. A rich body of fast-growing evidence is suggestive of the fact that gut bacterial richness and diversity are potential indicators of health and proper physiological function. Therefore, the transplanted fecal matter is generally obtained from a donor with a more favorable gut composition relative to the transplant recipient (169). The combination therapy–treated mice also demonstrated an increased T_reg_ population; decreased IL-2 levels in the CD4^+^, CD8^+^, and T_reg_^+^ cells; and regulated proinflammatory cytokines such as TNF-α and IL-17 (154). This study illustrated how a deeper understanding of the gut microbiota’s impact on immunosuppressant pharmacokinetics can serve as a valuable clinical datapoint capable of enhancing short-term as well as long-term transplantation success.

Current renal transplantation prophylaxis regimens involve an assortment of immunosuppressants such as mycophenolate mofetil, sirolimus, prednisone, and azathioprine, often with concomitant corticosteroid administration (132, 170, 171). Aside from immunosuppressant drugs, antimicrobials are an integral part of the standard post-transplantation pharmaceutical regimens to abate opportunistic infections in transplant recipients. To complicate matters even further, some immunosuppressant drugs such as tacrolimus and sirolimus can function as a macrolide antibiotic (132, 172). In fact, there is a significant degree of heterogeneity in the administered pharmaceutical regimens among the different patient cohorts reported in recent clinical studies investigating different human microbiotas in the context of renal transplantation (132, 141, 143). Therefore, it is unsurprising that the effects of immunosuppression on the microbiotas of transplant recipients can be confounded by the effects of antimicrobials as well as the cocktail of other concurrently prescribed drugs. For example, Rani and colleagues reported a shotgun metagenomics-based approach to study the urinary microbiota of kidney transplant patients, wherein the transplant recipients were treated with four doses of rabbit antithymocyte globulin for antibody induction, a calcineurin inhibitor (either tacrolimus or cyclosporin), mycophenolate (an antimetabolite immunosuppressant), and prednisone. The authors reported decreased bacterial diversity in the urinary microbiotas of transplant recipients, a finding in agreement with numerous investigations with comparable objectives (173). In the same report, the authors also expressed their concern surrounding the inadvertent selection of bacterial species capable of expressing antibiotic inhibition–bypassing metabolic pathways in the microbiotas of antibiotic-treated renal transplant recipients (173). Another group aimed to study the impact of immunosuppressant drugs on the gut microbiota–treated mice with prednisolone, mycophenolate mofetil, tacrolimus, a combination of these 3 drugs, everolimus, or water, respectively, for 2 weeks. As expected, the authors observed modifications in the composition of the microbiota in response to immunosuppression. Interestingly, single drug-treated mice and drug combination-treated mice demonstrated different modifications, suggesting that the effect of different drugs on the microbiota may not be additive in nature (174). Another study compared the rectal, oral, urinary, and blood microbiotas of renal allograft recipients before receiving the transplant as well as after 1 and 6 months of the same (175). Some of the patients participating in the study received antibody induction, whereas others did not. Over 90% of the patients undergoing antibody induction therapy were treated with Campath (alemtuzumab), a humanized anti-CD52 monoclonal antibody (137). Notable persistent changes in the pre-transplant and post-transplant microbiotas were observed. The most significant changes were observed between the pre-transplant and 1-month post-transplant microbiotas. An elevation in the numbers of *Firmicutes*, *Bacteroidetes*, *Proteobacteria*, and *Actinobacteria* was observed. This finding was indicative of the strong modulating effects the pre-, peri- and post-operative chemotherapeutic regimens such as immunosuppression have on patient microbiotas. The authors of the study also noted that the specific pre-transplant microbiota differences in certain patients were indicative of adverse post-transplantation consequences such as infections and even transplant rejection, thus corroborating the diagnostic, clinical, and therapeutic value of patient microbiota analyses (175). Studies exploring how the gut and, consequently, fecal microbiota could provide nephrologists with a treasure trove of actionable data points, enabling improved allograft health monitoring and maintenance, have also been reported (176, 177). Reports exploring the urinary microbiota in the context of renal transplantation have also yielded comparable insights (178, 179).

Over the past half-decade, explorations into the human urinary mycobiome and virome in the context of kidney transplantation have been reported. Wu and colleagues studied the urinary microbiotas of both male and female kidney transplant recipients suffering from chronic allograft dysfunction, a prelude to most graft failures (180, 181). The study reported notable urinary microbiota differences between chronic allograft dysfunction patients and their healthy counterparts. This study was also the first to explore the urinary mycobiome in graft dysfunction patients, instigating the research community engaged in human microbiota research to look beyond the bacteriome into other kingdoms (181). In another study, the authors performed a liquid chromatography–mass spectrometry/mass spectrometry (LC-MS/MS) analysis on urine samples sourced from a cohort of 142 kidney transplantation patients and normal healthy controls. The authors found 37 unique viruses such as Psittacid herpesvirus 1, *Spodoptera frugiperda*, Pseudocowpow virus, multiple nucleopolyhedrovirus, Japanese yam mosaic virus, and Cowpea mottle virus, 29 of which were never previously identified in human urine samples. Interestingly, some viral signatures were observed exclusively in the healthy control group, indicating the potential existence of viral commensals, some of which may even assist in immune adaptation (182). Another study detected multiple subtypes of BK polyomavirus, JC virus, and TTV in the urine samples of kidney transplant recipients. BK polyomavirus and JC virus infections are concerning for kidney transplant recipients as they can cause kidney and urinary tract infections, which can potentially lead to impaired renal function and even graft rejection (183). Although these viruses normally remain latent in the infected host, they can potentially reactivate in an immunosuppressed background. There is even evidence that BK polyomavirus infections undergo donor-to-recipient transfer during transplantation, thus further highlighting the clinical implications surrounding the aforementioned findings (184). Aside from the BK polyomavirus and JC virus, the Epstein–Barr virus also lies dormant inside the B cells of infected individuals with minimal symptomatic presentation, only to be reactivated after the onset of immunosuppression in kidney transplant recipients. The Epstein–Barr virus is responsible for approximately 90% of post-transplant lymphoproliferative disease cases, a well-known post-kidney transplant complication (185). Overall, much remains to be understood about the viromes of the different human microbiotas. Furthermore, since bacteriophages are the most prevalent component of the human virome, deciphering the complex bacteriophage–bacteria interaction dynamics of the different microbiotas may galvanize the development of bacteriophage cocktails, which can supplement or, in some cases, replace existing antibiotic therapy in immunosuppressed patients (186).

Pharmaceutical interventions are not the only factors triggering changes in the human microbiotas. A recent study demonstrated post-transplantation gut dysbiosis in murine kidney transplantation models in the absence of any form of pharmaceutical intervention (131). In the study, the authors demonstrated the allograft-protective properties of fiber-rich diets and short-chain fatty acids (sodium acetate or sodium butyrate) in murine kidney transplantation models, suggesting the alloimmunity-retarding role of gut-derived acetate. Since acetate exhibits preferential binding to metabolite-sensing G-protein coupled receptors such as GPR43, the alloimmunity-retarding role of gut-derived acetate was further illustrated by the ablation of the dietary intervention–promoted survival advantage in GPR43^−/−^ mice. This study demonstrated the immunomodulatory capabilities of the gut microbiota in response to dietary interventions, therefore providing evidence in favor of combination therapies over strict pharmaceutical regimens for allograft maintenance (131). In fact, dietary modifications are known to have a modifying effect on the health of kidney transplant recipients (187–189). The Mediterranean and DASH (Dietary Approaches to Stop Hypertension) diets have been demonstrated to be the most beneficial dietary patterns for renal transplant recipients due to their emphasis on increasing fresh plant-based food intake as well as decreasing processed food and meat intake (187). For example, a recent single-center cohort-based clinical study involving 632 adult kidney transplant recipients reported in 2020 by Gomes-Neto and colleagues demonstrated the improved kidney function outcomes in renal transplant recipients following the Mediterranean diet plan (188). In fact, the mechanistic underpinnings underlying changes in the gut microbiota due to dietary interventions such as the Mediterranean and DASH diets as well as their impact on host physiology and various disease processes are active areas of investigation (190–194). The effect of specific dietary interventions on the various microbiotas of renal transplant recipients and its impact on allograft maintenance, however, remains to be investigated.

Kidney transplant recipients have been documented to be at an up to fourfold higher risk of cancer and cancer-associated death than healthy individuals (195). Observational evidence also suggests an association between CKD and elevated cancer risk as well as unsatisfactory cancer outcomes (185). Several risk factors such as donor-transmitted malignancies, donor type (living vs. deceased), recipient age, dialysis time before transplantation, a history of cancer prior to transplant, viral infections, and the immunomodulatory effects of immunosuppressive therapy have been identified to elevate cancer risk in kidney allograft recipients (195–197). Interestingly, the most common type of cancer observed in kidney transplant recipients is skin cancer. The most reported forms of skin cancer reported in renal transplant recipients include basal cell carcinoma, malignant melanoma, cutaneous squamous cell carcinoma, and Kaposi sarcoma. In fact, renal transplant recipients are at an up to 250 times higher risk of developing squamous cell carcinoma than the general population (185, 198). The pathogenesis of skin carcinoma is precipitated by a constellation of risk factors, as discussed above. Of course, the elevated risk of skin cancer is applicable to all solid organ transplantation scenarios as they all require immunosuppression for graft maintenance (198). Kidney transplant recipients are also at an up to seven-fold higher risk of developing renal cell carcinoma, the most common form of kidney cancer (185). A recent study aimed to temporally characterize the treatment-related compositional changes occurring over the course of checkpoint inhibitor therapy by analyzing the stool samples of metastatic renal cell carcinoma patients undergoing therapy. Checkpoint proteins such as PD-L1 are a class of proteins that bind to T-cell surface receptors to downregulate their activity and prevent damage to a body’s own cells. Checkpoint inhibitor therapy is a type of cancer immunotherapy that targets and binds to key immune checkpoint proteins, thereby blocking T-cell inhibitory responses, restoring immune function, and consequently directing the immune system against malignant cells (199–201). The authors reported an association between higher microbial diversity and optimistic treatment outcomes, a finding in agreement with observations made in studies investigating allograft maintenance outcomes in the context of different human microbiotas (141, 143, 202–204). The metagenomic implications of different chemotherapeutic as well as dietary interventions in renal cell carcinoma patients is an area of active investigation. A recent clinical study reported that gut microbiota composition is influenced by the administration of antibiotics and tyrosine kinase inhibitors, thereby impacting the success of renal cell carcinoma immune checkpoint inhibitor therapy (205). In the same year, a prospective randomized study demonstrated the probiotic supplementation–induced gut microbiota modulation of metastatic renal cell carcinoma patients receiving vascular endothelial growth factor tyrosine kinase inhibitor therapy, the primary line of treatment for patients with advanced renal cell carcinoma. Although the study failed to demonstrate anticancer effects in human subjects, it revealed interesting insights such as the increased relative abundance of *A. muciniphila, B. caccae, F. prausnitzii*, and *B. intestinihominis* in the patient group responding well to immunotherapy. Since there are currently no biomarker-based approaches to metastatic renal cell carcinoma treatment selection, these insights may serve as starting points for future investigations focusing on the identification and validation of microbial signature–based biomarkers (206).

The gut microbiota is, however, not the only human microbiota under active investigation in the context of renal cell carcinoma. Heidler and colleagues were the first to investigate and contrast the renal microbiota of healthy and tumor-bearing kidney parenchyma using biopsy samples obtained from patients undergoing laparoscopic nephrectomy for renal carcinoma. The authors observed a heterogenous distribution of microorganisms colonizing the benign and malignant tissue. For example, *Thermicanus aegyptius, Anaerococcus nagyae, Leuconostoc garlicum, Neisseria bacilliformis, Corynebacterium vitaeruminis, L. mesenteroides, and Ethanoligenens harbinense* were observed to only colonize the benign tissue whereas *Mycoplasma vulturii, Phaeocystis antarctica, Spirosoma navajo, Euglena mutabilis*, and *Cyanophora paradoxa* were observed to exclusively colonize the malignant tissue (207). Another group studying the renal tumor microbiota reported renal tumors to have more diverse microbiotas as compared to benign tissue (208). The discovery of exotic microbiotas such as the renal microbiota directly contradicts the traditional overestimates of the sterility of various human organ systems. The urinary bladder and, consequently, urine were also once considered to be sterile in healthy individuals but have since been demonstrated to host the urobiome (the urinary microbiota), the alterations in which have been associated with different urinary pathologies (179, 181, 186, 207). Emerging evidence across multiple domains is indicative of the existence of numerous complex microbiotas in various locations of the body that are in constant bidirectional crosstalk with each other as well as our organ systems. For example, the crosstalk between the gut and kidney, referred to as the gut–kidney axis, has been implicated in a wide range of renal disorders such as hypertension, nephrolithiasis, immunoglobulin A (IgA) nephropathy, and CKD (209, 210).

**Figure 3:**
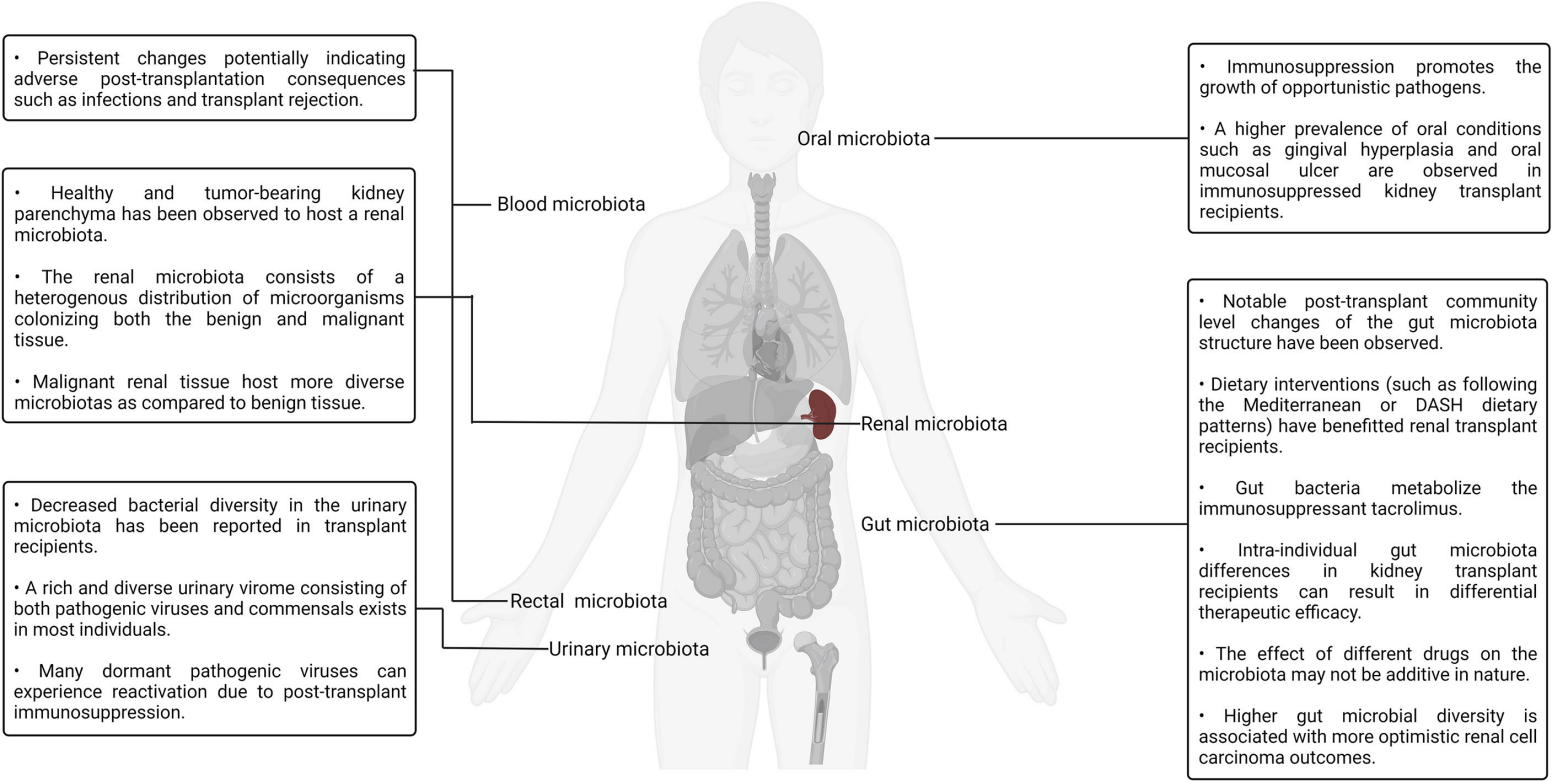
A visual summary of some of the key highlights discussed in the context of kidney transplantation and the human microbiotas.

The role of the various human microbiotas, especially the gut microbiota, as a biomarker and modulator of therapeutic outcome has been well established both in the context of renal transplantation and cancer. Numerous groups have also viewed the gut microbiota as a therapeutic target and attempted to modulate it using dietary as well as prebiotics- and probiotics-based interventions. A more direct approach to targeted gut modulation, that is, FMT, is a promising new category of therapeutic interventions targeting gut dysbiosis that may yield superior clinical outcomes as compared to the current alternatives. For example, FMT-based treatment has been documented to be a superior line of treatment for recurrent *C. difficile* infection (89.6% remission rate) as compared to conventional antibiotic-based therapeutic interventions typically involving metronidazole, vancomycin, rifaximin, and fidaxomicin (211). Several clinical trials evaluating the viability of fecal transplantation and its impact on renal transplantation as well as cancer outcomes have been conducted, are currently underway, or are slated to start soon. Translating insights derived from murine model–based research, as well as human clinical trials into robust and effective standardized clinical therapeutic protocols, will require both a depth-first and breadth-first expansion of microbiota surveillance under different pharmaceutical, physiological, pathological, and genetic backgrounds (212). Expanding the scope of studies beyond metagenomics to include metabolomic, proteomic, and transcriptomic insights will also help in elucidating the mechanistic underpinnings of the host–microbiota interaction dynamics. A visual summary of some of the key highlights discussed in this section is illustrated in **Figure 3**.

## Lung Transplantation

An unsubstantiated presupposition pertaining to the sterility of the lungs was extant for a considerably long period of time till the first culture-independent report of the healthy lung microbiota in 2010 conclusively debunked its absence (9, 213). Soon thereafter, another group reported a study in which the microbial population colonizing the transplant patient respiratory tract was comprehensively characterized using unbiased high-density sequencing (214). In the study, the authors carried out bacterial (16S rRNA) and fungal (internal transcribed spacer) gene sequencing to identify the organisms present in bronchoalveolar lavage and oropharyngeal wash samples sourced from transplant recipients as well as healthy controls. Bronchoalveolar lavage is a procedure in which a saline solution is passed through a bronchoscope to wash the airways and extract a fluid sample from the lungs for testing (215). Notable differences in the community structure and composition between the healthy and transplanted lungs were observed. Compared to the control samples, lung transplant recipient–sourced bronchoalveolar lavage was observed to have higher bacterial loads, lower microbial richness as well as diversity, and more taxonomically distinct populations. The oropharyngeal wash samples were rich in bacterial taxa that are typically associated with the oral cavity such as *Streptococcus*, *Prevotella*, *Veillonella*, *Porphyromonas*, *Neisseria*, and *Rothia*. Although the bacterial profiles found in bronchoalveolar lavage and oropharyngeal wash samples had significant overlaps, specific bacterial populations were observed to dominate the lung transplant recipient–sourced samples. The presence of *Candida* and *Aspergillus* was also observed in bronchoalveolar lavage samples. Although this study provided several interesting insights regarding the transplanted lungs and their resident microbiota, the authors did not establish any causal relationships between graft failure, bronchiolitis obliterans syndrome, and the presence or absence of individual genera/species in the lung microbiota (214). Bronchiolitis obliterans syndrome, a form of chronic lung allograft rejection, is among the most common noninfectious transplant–related complications. It is characterized by bronchiolar smooth muscle hypertrophy, peribronchiolar inflammatory infiltrates, mucus accumulation in the bronchiolar lumen, bronchiolar scarring, and even complete bronchial lumen occlusion in some cases (216).

Other studies have reported findings in agreement with that reported by Charlson and colleagues. For example, one group analyzed the bacterial sequences in the bronchoalveolar lavage fluid samples of four lung transplant recipients and found that the transplanted lungs had a higher richness as well as diversity of bacterial sequences. Furthermore, the healthy lung microbiota was mostly predominated by the members of the phylum *Proteobacteria* (class *Gammaproteobacteria*) and *Firmicutes* whereas the transplanted lung microbiota was predominated by the members of phylum *Proteobacteria* (class *Betaproteobacteria*) (217). Another study comparing bronchoalveolar lavage samples sourced from lung transplant recipients with and without bronchiolitis obliterans syndrome as well as healthy controls (individuals with no history of lung transplantation) revealed that transplanted patients had notable differences in the community composition but similar bacterial diversities when compared to the healthy controls. Furthermore, according to the findings of the study, lung transplant recipients are more likely to harbor *Staphylococcus, Pseudomonas*, *Proprionobacterium*, and *Veillonella*, which were the typical genera (218, 219).

Although post-transplant changes have been established by several reports, the underlying mechanics of the lung microbiota changes occurring post-transplant is still under investigation. In addition to surgical factors such as vagal denervation (resulting in compromised afferent stimulation and, consequently, aberrant cough reflex), the post-transplant administration of immunosuppressive and antimicrobial drugs are potential culprits. Based on the notable effects antimicrobial administration has on the gut microbiota, it is natural to assert that antimicrobials have a notable impact on the microflora inhabiting the lung microbiota (220, 221). In a study aiming to understand the comparative relative influence of gut and lung bacteria on the baseline lung immune tone, ceftriaxone was demonstrated to reduce the relative abundance of *Proteobacteria* while at the same time increasing the relative abundance of *Firmicutes* in mice (222). On the other hand, another study aiming to decipher the influence of azithromycin, a macrolide drug possessing immunomodulatory and antibacterial properties, on lung allograft rejection as well as the post-transplant lung microbiota found no notable effects of the drug on the post-transplant lung microbiota community structure, composition, or diversity (223, 224). Similarly, much is not currently known about the impact of systemic immunosuppression on the lung microbiota as it has not been reported directly. It is an active area of investigation. Even though our understanding of the lung microbiota is currently incomplete, it is sufficient to derive clinically translatable insights. A recent study analyzed bronchoalveolar lavage samples sourced from lung transplant recipients a year after the procedure and found that the bacterial biomass of the lung microbiota was a strong predictor of chronic lung allograft dysfunction development as well as death (225, 226). Chronic lung allograft dysfunction refers to a collection of pathological conditions that prevent a transplanted lung to achieve or maintain typical function.

The study conducted by Charlson and colleagues back in 2012, as already discussed, presented evidence pertaining to the existence of a distinct fungal populace in the lung also referred to as the mycobiome. Two years later, the same group published a study focused on characterizing clinically relevant fungal lineages present in the oropharyngeal wash as well as bronchoalveolar lavage samples of healthy controls, HIV+ patients, and lung transplant recipients, thereby establishing a gradient of progressively increasing lung impairment for comparative purposes (227). The increasing severity of pulmonary and immunologic deficits in the patients was observed to be accompanied by an elevated representation of *Candida*, *Aspergillus*, and *Cryptococcus.* Furthermore, it was also observed that *Candida*-rich oropharyngeal communities demonstrated a positive covariance with members of the *Streptococcus mitis* group, potentially indicating cross-kingdom interactions. *S. mitis* is a group of bacteria, many of which are recently classified, poorly characterized, and pathogenic. They natively colonize the human oral cavity, nasopharynx, and GI tract (227–229). In a recent study, a group applied deep-sequencing to analyze the microbial biofilms present on endobronchial stents (a silicone/steel tube that keeps the bronchial tubes open in patients affected by bronchial stenosis) in a patient cohort predominantly consisting of lung transplant recipients (230). The authors reported *Candida* spp. and *Aspergillus* to have the highest and second-highest abundance in the biofilms, respectively. Aside from fungal taxa, the existence of several bacterial taxa such as *Corynebacterium* (most common), *Staphylococcus*, *Pseudomonas*, *Streptococcus*, and *Prevotella* was also noted by the authors. Furthermore, some evidence indicating fungal–bacterial covariation was also observed in this study (230).

In addition to the mycobiome, the lung hosts a diverse virome and phageome that, alongside the mycobiome and bacteriome, have potential implications for lung transplantation success and the overall lung immunity. Several studies exploring the lung virome have been reported in the recent past. For example, in one such single-center, prospective, longitudinal study, the authors analyzed the viral communities present in transplanted lungs and detected the presence of community-acquired respiratory viruses such as influenza A, parainfluenza, and human rhinovirus. The detected viruses were, however, not associated with transplant rejection (231). In another study, a group aiming to characterize the post-transplant respiratory tract DNA virome analyzed the viral communities present in lung transplant recipient–sourced bronchoalveolar lavage as well as oropharyngeal wash samples (232). They then compared the same with bronchoalveolar lavage and oropharyngeal wash samples obtained from healthy as well as HIV+ individuals. Although anelloviruses were detected in all the samples, the lung transplant recipient–sourced samples hosted a significantly richer and more diverse population consisting of multiple anellovirus variants. In fact, lung transplant recipient–sourced samples had a 56× higher content of anelloviruses. Papilloma viruses, herpes viruses and bacteriophages were also detectable, albeit at much lower levels in comparison to the anellovirus signal. Interestingly, large numbers of bacteriophages were detected in the transplant subject–sourced samples compared to a very limited number of mammalian viruses (anelloviruses being the only exception). It is, however, likely that the confounding effects of the antiviral prophylaxis, transplant subjects routinely administered, contributed to this asymmetrical distribution of bacteriophages and mammalian DNA viruses. Many sequences yielding no hits on the National Center for Biotechnology Information (NCBI) database were also obtained during the metagenomic analysis, indicating the existence of novel bacteriophage and mammalian virus strains in the post-transplant lung virome. High annellovirus burdens were also found to be correlated to bacterial dysbiosis in the transplant recipient–sourced bronchoalveolar lavage samples, suggesting cross-kingdom interactions (232).

A notable number of studies report the notable predominance of *Anelloviridae* (such as the TTV) in lung transplant recipient–sourced bronchoalveolar lavage and oropharyngeal wash samples (223, 232–234). In fact, the repeated occurrence of this observation has prompted discussions into the possibility of anellovirus-based immune status biomarkers (235). Furthermore, the interest surrounding anelloviruses extends beyond lung transplantation and has been discussed in the context of overall pulmonary health and chronic respiratory diseases (236). A comprehensive study investigating viral post-transplant temporal dynamics reported the bidirectional movement of viral populations between donor and recipient lungs, implicating that the commensal viral communities constituting the virome of a lung allograft are transplanted alongside the organ. Furthermore, alongside anelloviruses (the most abundant), other viral families such as herpesviruses, parvoviruses, polyomaviruses, and even bacteriophages were also detected in the study. Interestingly, a different study reported the presence of a novel viral family termed *Redondoviridae* in human bronchoalveolar lavage and oropharyngeal samples (237). What was even more surprising was that the frequency at which the members of the family *Redondoviridae were* detected in the samples was second only to the detection frequency of *Anelloviridae*. Furthermore, two were found to co-occur at statistically significant levels. Given the documented elevation of the anellovirus load in post-transplant lung allografts, the rich commensal anellovirus population constituting the healthy human virome, and our limited understanding of the human virome, studies focusing on building a deeper understanding of post-transplant viral dynamics is the need of the hour (233, 234, 237–239). The existence of bacteriophages in the lung virome is also noteworthy since they can have a modulatory role on the resident bacteria of the lung microbiota that can be leveraged in clinically translatable directions (14, 232). Studies describing multimodal therapeutic interventions involving antibiotics as well as bacteriophages to treat the multidrug-resistant pathogenic strains of *P. aeruginosa* and *Burkholderia dolosa* have reported optimistic therapeutic outcomes and no adverse effects (240, 241).

The use of probiotics-based therapeutic interventions in the amelioration of lung diseases has also been explored by several groups. For example, the oral supplementation of *E. faecalis FK-23* has been documented to attenuate allergic airway inflammation and Th17-cell development in mouse allergic asthma models (242). The use of probiotics has also been discussed in the context of severe acute respiratory syndrome coronavirus 2 (SARS-CoV-2) prevention and treatment (243). However, the prospect of probiotic consumption by immunocompromised patients such as lung transplant recipients is deeply intertwined with concerns pertaining to its safety. In their case report, Luong and colleagues describe how a concerning elevation in the number of *Lactobacillus* infections was recorded in a hospital after *L. acidophilus/helveticus* was replaced with *L. rhamnosus* GG as the principal constituent of the prophylactic probiotic supplementation given to heart and lung transplant patients for *C. difficile–*associated diarrhea. In fact, the routine administration of probiotic formulations to patients was subsequently discontinued due to the dearth of convincing evidence regarding its benefits as compared to the established downsides (244). The literature surrounding the administration of FMT to restore the lung microbiota is not well developed. One group reported a study in which a patient-derived multidrug-resistant *Pseudomonas* isolate was engrafted into humanized murine lungs following which host cytokine responses, as well as the notable post-engraftment lung and gut microbiota changes, were studied. Gut FMT was demonstrated to have a therapeutic effect on multidrug-resistant bacterial lung infections (245). More elaborate studies in this direction are anticipated in the years to come.

**Figure 4:**
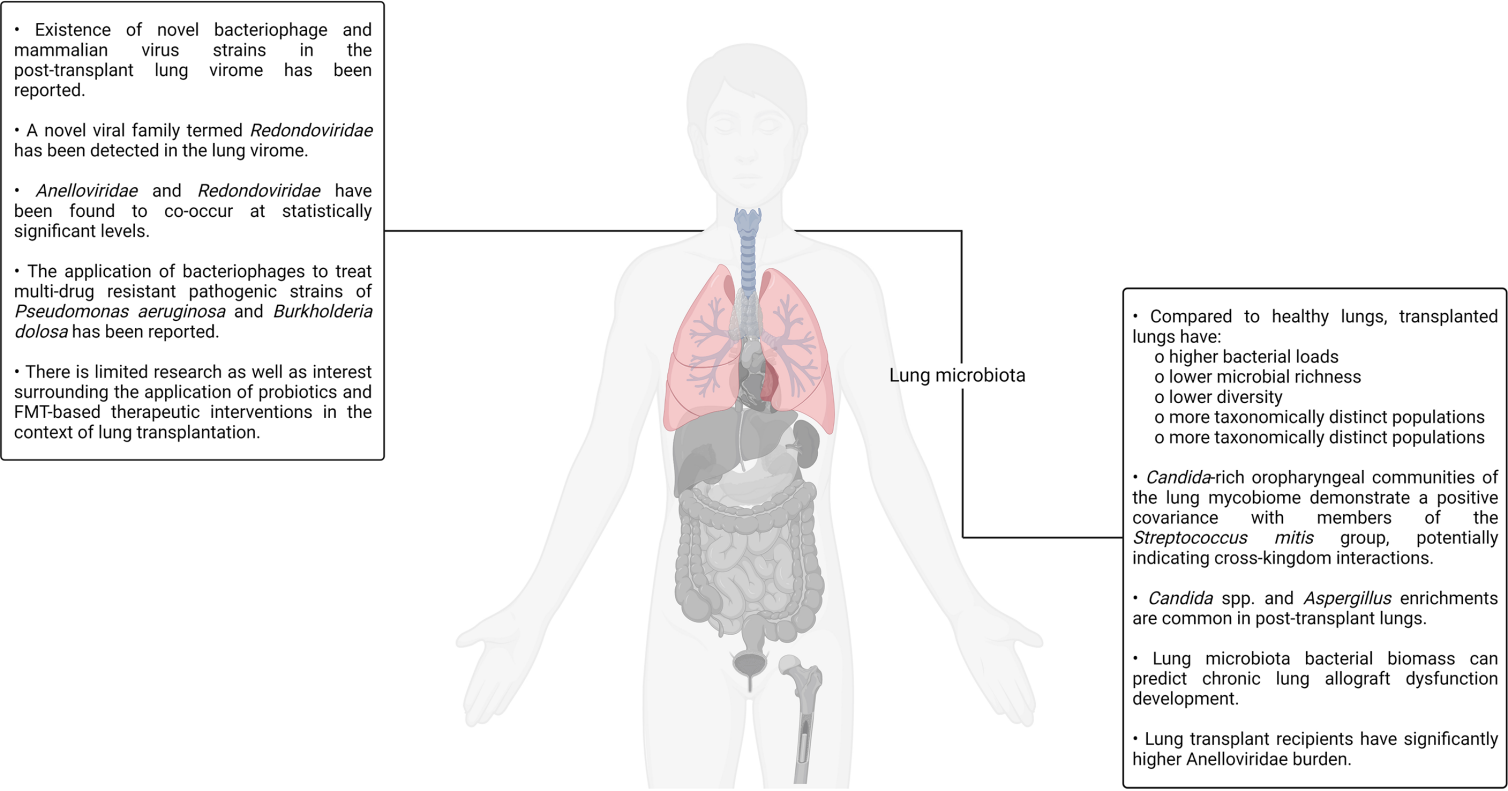
A visual summary of some of the key highlights discussed in the context of lung transplantation and the human microbiotas.

A recent study categorized post-transplant lung microbiotas into four distinct compositional states determined by an aggregate of factors including anellovirus loads, respiratory function, and even host immune responses (246). A balanced compositional state, characterized by diverse bacterial communities with moderate viral loads, indicates a healthy lung microbiota. On the other hand, dysbiotic compositional states, characterized by depleted or pathogen-dominated microbiotas, are indicative of elevated immune activity and infection risk, depressed pulmonary function, and increased lung allograft rejection risk (246). This study delineates the intimate yet complex association between respiratory health, the resident lung microbiota, the bacteriome–virome–host physiology axis, human lung function, respiratory health, and most importantly, post-transplantation clinical stability of lung allografts. Recent advancements in next-generation sequencing technologies and metagenomic workflows have galvanized the exploration of challenging microbiotas such as that of the lung (247). With further technological and methodological advancements, more and more insightful reports exploring the lung microbiota in health, disease, and various transplantation scenarios can be expected to surface in the upcoming years. A visual summary of some of the key highlights discussed in this section is illustrated in **Figure 4**.

## Liver and Heart Transplantation

Aside from the transplantation scenarios discussed above, organs such as the heart and liver are also transplanted across the globe. There has also been a multitude of reports exploring a diverse range of perspectives involving each of these transplantation scenarios in the context of human microbiotas. For example, a recent study analyzing the pre- and post-transplant fecal microbiotas of a cohort of patients consisting of heart–kidney, heart–liver, heart–liver–kidney, or just liver transplant recipients has been reported (248). This study aimed to compare fecal samples sourced from different heart transplant recipients with those sourced from liver transplant recipients as well as healthy controls to better understand the metagenomic and metabolomic changes occurring across the transplantation timeline. The study revealed that heart transplantation patients displayed a lower within-sample microbial diversity but a higher frequency of *Lactobacillus*, *Enterococcus*, and *Faecalicoccus* detection as compared to healthy controls. Among the different transplant recipients, liver transplant recipients had the lowest within-sample microbial diversity and marked losses of normal bacterial taxa. Moreover, both heart transplant and liver transplantation recipients demonstrated notable differences in the relative abundances of butyrate-producing anaerobic bacterial species such as *Lachnospiraceae* and *Ruminococcaceae*, with heart transplant recipients having significantly higher abundances. Aside from the discussed observations, this study also demonstrated how the nature of the changes occurring in the gut microbiota as a response to organ transplantation can distinctly vary based on the specific organ being transplanted (248). Wider and deeper studies in this direction can be expected in the upcoming years. In another study, an immunocompromised pediatric heart transplant recipient received FMT to treat recurrent *C. difficile* infection. The recipient reported a restoration of healthy microbial diversity without any concomitant transplant complications or infection relapses (249). This was a remarkable report as the FMT recipient was the youngest immunocompromised patient to undergo the procedure, although successful FMT procedures on other pediatric cardiac transplant patients have been reported earlier (250). Although there is some evidence demonstrating the safety and efficacy of FMT, the consistency of the positive outcomes needs to be validated in larger groups ideally spanning multiple geographies and ethnicities.

Liver transplantation has also been documented to significantly alter the gut microbiota. A group compared the fecal microbiota of healthy liver transplant recipients with that of liver transplant recipients presenting with abnormal liver function. All liver transplant recipients displayed an elevated relative abundance of opportunistic pathogens (*Klebsiella*, *Escherichia*/*Shigella*) as compared to healthy controls. However, the interesting point of difference among the liver transplant recipients was that liver transplant recipients presenting with abnormal liver function had a notably lower relative abundance of butyrate-producing gut bacteria such as *Lachnospiraceae*, *Odoribacteraceae*, and *Clostridiaceae*, as compared to their healthy counterparts. This study once again reinforced the importance of short-chain fatty acids as well as their producers in maintaining gut health across a variety of different transplantation scenarios (251). A different study analyzed fecal samples sourced from a cohort of liver transplant recipients with a history of non-alcoholic fatty liver disease, among which 71% presented with a disease recurrence. The authors found evidence suggestive of the protective roles of *Akkermansia*, *Firmicutes*, and *Bifidobacterium* as well as the pathogenic roles of *Fusobacteria* and *Bacteroidetes* (252).

Aside from the bacteriome, the plasma virome also has a documented role in determining liver transplantation outcomes (253). A recent study analyzing the plasma virome of liver transplant recipients both pre- and post-liver transplant reported an *Anelloviridae* bloom dominating the immunosuppressed post-transplant plasma virome that was accompanied by several complications. The potential of *Anelloviridae*-based diagnostic markers has been discussed in the context of HSCT and lung transplantation. The findings of this study also demonstrate the relevance of the same for the detection of post-liver transplant complications. Interestingly, all human pegivirus–positive liver transplant recipients were found to be alive half a decade after undergoing the transplantation procedure, suggesting potentially beneficial, positive patient outcome–promoting immune system modulation by the virus (253). However, further investigation in this regard is required before robust conclusions can be drawn.

**Figure 5:**
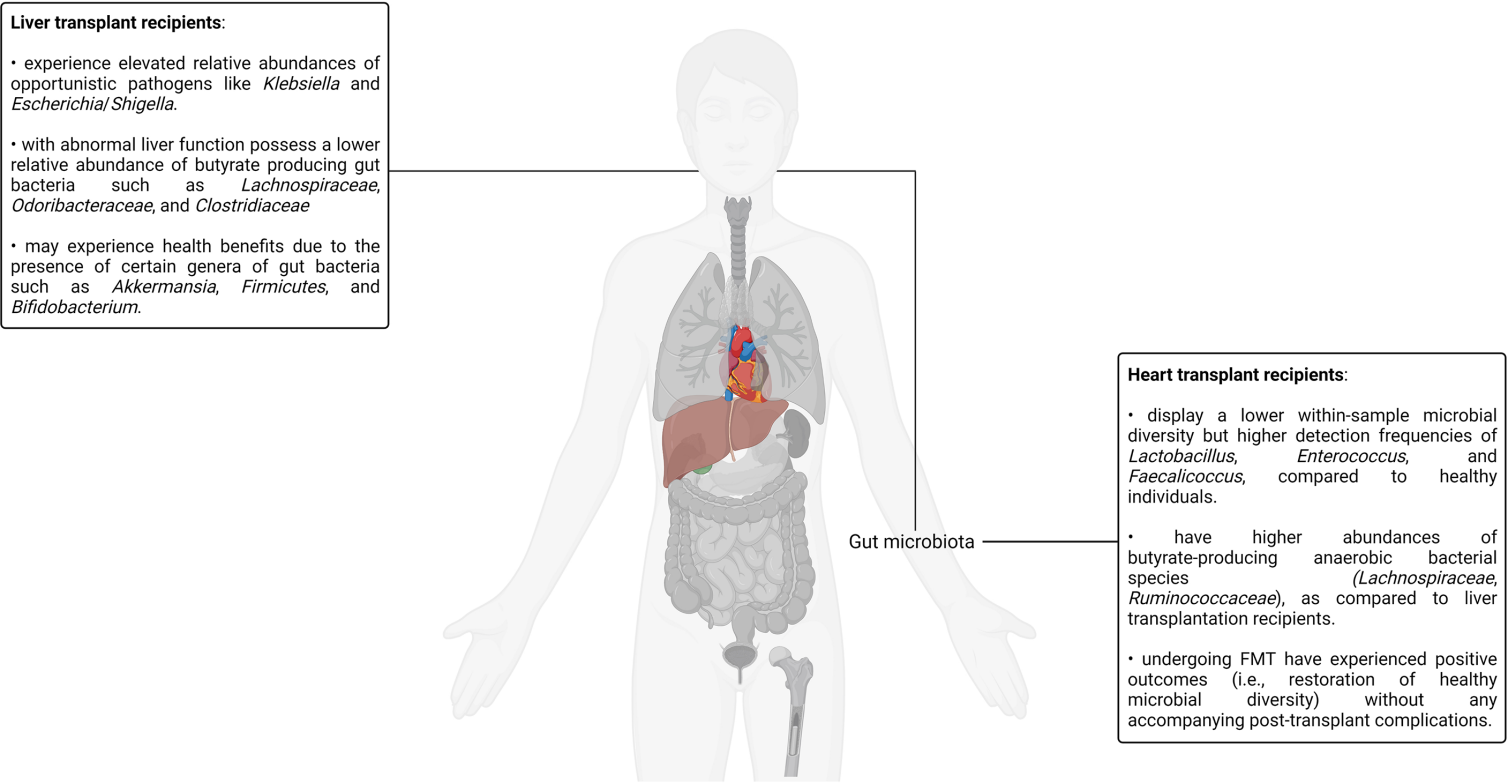
A visual summary of some of the key highlights discussed in the context of liver as well as heart transplantation and the human microbiotas.

The use of probiotics has also been investigated in the context of liver transplantation. A randomized, double-blind, and placebo-controlled clinical trial placed adult liver cirrhosis patients on a four-strain probiotic supplementation regimen consisting of *Lactococcus lactis*, *L. casei*, *L. acidophilus*, and *Bifidobacterium bifidum*, once daily before breakfast until the commencement of the liver transplantation procedure (254). Compared to the placebo control group, the probiotic-supplemented liver transplant recipients reported substantially lower post-transplant infection rates as well as improved biochemical markers of allograft function such as bilirubin concentration, and (aspartate and alanine) aminotransferase activity. However, in this study, probiotic administration did not seem to have any notable impacts on postoperative mortality (254). More studies exploring the impact of probiotics as well as prebiotics on liver transplant outcomes need to be conducted before the scientific and medical community can reach a unilateral consensus pertaining to the benefits of the same. A visual summary of some of the key highlights discussed in this section is illustrated in **Figure 5**.

## Conclusion

This review has discussed the effect of five different transplantation scenarios, that is, HSCT, renal transplantation, lung transplantation, liver transplantation, and heart transplantation on the various human microbiotas. Three transplantation scenarios, that is, HSCT, renal transplantation, and lung transplantation, have been discussed in depth. HSCT is the only form of clinically standardized stem cell transplantation that is routinely carried out for a variety of different malignant and non-malignant conditions. Furthermore, studying HSCT in the context of the human microbiotas gives us a rare opportunity to study cancer, stem cells, and the human microbiotas in a contextually relevant, mutually non-exclusive, and semantically associated framework. Renal transplantation is the oldest and most prevalent form of solid organ transplantation. Naturally, many comprehensive studies exploring renal transplantation and its association with various human microbiotas have been reported over time. Therefore, the body of research exploring the intersection of the human microbiotas and kidney transplantation is among the most mature in all transplantation scenarios. Therefore, exploring kidney transplantation in the context of the human microbiotas serves as a good primer for similar investigations related to other transplantation scenarios. Finally, the lungs are the only fully transplantable organ with their own resident, self-contained microbiota as well as a well-developed immune system that effectively neutralizes a constant barrage of antigens, pathogens, and other immunological challenges. Furthermore, the lung microbiota is not very well characterized due to its significantly lower biomass as compared to the gut microbiota and the persistent challenges involved in obtaining contamination-free samples (218). Therefore, a deeper look into lung transplantation and its impact on the lung microbiota may provide us with unique insights pertaining to host–microbiota interactions and their bidirectional impact on transplantation as well as transplantation outcomes. Although an investigation into the impacts of heart and liver transplantation on the human microbiotas is by no means less important or less insightful, most of the trends and overarching patterns observed in the context of HSC, kidney, and lung transplantation are consistent with that of heart and liver transplantation. A deeper exploration of these topics is nevertheless warranted.

This review discusses the effects of HSC and organ transplantation on various human microbiotas and how our understanding of the same can be leveraged in clinically translatable directions. However, the relevance of the human microbiotas is not limited to transplantation, nor are they limited to the specific microbiotas discussed in this review. For example, uterine transplantation and its impact on the vaginal microbiota is an emerging area of investigation. The vaginal microbiota is an important, intricate, and dynamic human microbiota that changes at different points of a woman’s menstrual cycle as well as her entire life. Physiological, psychological, and situational factors such as hormonal levels, pregnancy status, sexual activity, douching, stress levels, race, and regional disparity are also known to impact the vaginal microbiota (8). Uterine transplantation is a surgical procedure involving the transplantation of the uterus, cervix, surrounding connective tissue, blood vessels, and a cuff of the vagina from a healthy female donor to a female recipient suffering from absolute uterine factor infertility (255). Absolute uterine factor infertility is a clinical condition in which a woman is infertile due to the anatomical or functional deficit of a uterus. Absolute uterine factor infertility can both be congenital and acquired (removed during a hysterectomy). Although uterine transplantation is in its nascency with only a handful of cases reported worldwide, it is gaining attention in the medical community (255, 256). The ocular microbiota is another example of a unique microbiota that has been reported to be interrelated to a variety of ophthalmic diseases such as age-related macular degeneration, autoimmune uveitis, glaucoma, and several others (257). The recent discovery of even more exotic human microbiotas such as the seminal and penile microbiota, hepatic microbiota, and cerebrospinal fluid virome opens up new frontiers of metagenomic exploration (8, 11, 258–260). With the accumulation of more and more studies, the complex interconnectivity between different organ systems and the various human microbiotas is becoming increasingly apparent. How these insights will translate into beneficial diagnostic and therapeutic interventions in the future is yet to be seen.

## Author Contributions

TS wrote the original draft of the manuscript under the guidance of RT. RT reviewed and edited the draft. All authors read and approved the final draft of the manuscript.

## Conflict of Interest

The authors declare that the research was conducted in the absence of any commercial or financial relationships that could be construed as a potential conflict of interest.

## Publisher’s Note

All claims expressed in this article are solely those of the authors and do not necessarily represent those of their affiliated organizations, or those of the publisher, the editors and the reviewers. Any product that may be evaluated in this article, or claim that may be made by its manufacturer, is not guaranteed or endorsed by the publisher.
